# Comparison of ultrasound−based ADNEX model with magnetic resonance imaging for discriminating adnexal masses: a multi-center study

**DOI:** 10.3389/fonc.2023.1101297

**Published:** 2023-04-24

**Authors:** Yanli Hu, Bo Chen, Hongmei Dong, Bo Sheng, Zhibo Xiao, Jia Li, Wei Tian, Furong Lv

**Affiliations:** ^1^Department of Ultrasonography, Chongqing Health Center for Women and Children, Chongqing, China; ^2^Department of Ultrasonography, Women and Children’s Hospital of Chongqing Medical University, Chongqing, China; ^3^Department of Radiology, The First Affiliated Hospital of Chongqing Medical University, Chongqing, China; ^4^Department of Ultrasonography, The First Affiliated Hospital of Chongqing Medical University, Chongqing, China; ^5^Department of Radiology, Women and Children’s Hospital of Chongqing Medical University, Chongqing, China; ^6^Department of Radiology, Chongqing Health Center for Women and Children, Chongqing, China

**Keywords:** adnexal mass, ovarian cancer, magnetic resonance imaging, adnex, ultrasound

## Abstract

**Objectives:**

The ADNEX model offered a good diagnostic performance for discriminating adnexal tumors, but research comparing the abilities of the ADNEX model and MRI for characterizing adnexal tumors has not been reported to our knowledge. The aim of this study was to evaluate the diagnostic accuracy of the ultrasound-based ADNEX (Assessment of Different NEoplasias in the adneXa) model in comparison with that of magnetic resonance imaging (MRI) for differentiating benign, borderline and malignant adnexal masses.

**Methods:**

This prospective study included 529 women with adnexal masses who underwent assessment *via* the ADNEX model and subjective MRI analysis before surgical treatment between October 2019 and April 2022 at two hospitals. Postoperative histological diagnosis was considered the gold standard.

**Results:**

Among the 529 women, 92 (17.4%) masses were diagnosed histologically as malignant tumors, 67 (12.7%) as borderline tumors, and 370 (69.9%) as benign tumors. For the diagnosis of malignancy, including borderline tumors, overall agreement between the ADNEX model and MRI pre-operation was 84.9%. The sensitivity of the ADNEX model of 0.91 (95% confidence interval [CI]: 0.85–0.95) was similar to that of MRI (0.89, 95% CI: 0.84–0.94; *P*=0.717). However, the ADNEX model had a higher specificity (0.90, 95% CI: 0.87–0.93) than MRI (0.81, 95% CI: 0.77–0.85; *P*=0.001). The greatest sensitivity (0.96, 95% CI: 0.92–0.99) and specificity (0.94, 95% CI: 0.91–0.96) were achieved by combining the ADNEX model and subjective MRI assessment. While the total diagnostic accuracy did not differ significantly between the two methods (*P*=0.059), the ADNEX model showed greater diagnostic accuracy for borderline tumors (*P*<0.001).

**Conclusion:**

The ultrasound-based ADNEX model demonstrated excellent diagnostic performance for adnexal tumors, especially borderline tumors, compared with MRI. Accordingly, we recommend that the ADNEX model, alone or with subjective MRI assessment, should be used for pre-operative assessment of adnexal masses.

## Introduction

Adnexal malignancy is an uncommon, life-threatening gynecological tumor with a high recurrence rate and low survival rate (1). It is usually detected at an advanced stage, contributing to the low 5-year survival rate. However, when detected in the early stage, the 5-year overall survival rate is more than 90% (2), such as borderline ovarian tumors (BOTs) survival is 95% at 5 years (3). Therefore, accurate early diagnosis of adnexal tumors is not only crucial for improving patient survival by applying appropriate treatments, which differ according to the status of tumor (4–6), but also important for the young female patients who want to preserve their fertility potential (7). Benign masses can be observed *via* follow-up or locally excised *via* laparoscopic surgery and BOTs could even adopt a strategy of fertility-sparing surgery because of its excellent reproductive outcome and long-term survival (7), whereas malignant masses must to properly stage and debulking surgery performed by a gynecological oncologist (8).

Imaging techniques, including transvaginal ultrasound and magnetic resonance imaging (MRI), are important tools for the preoperative evaluation of adnexal tumors (5, 6, 8). Although transvaginal ultrasound is a preferred method for the detection of adnexal masses, the value of this method for the diagnosis of adnexal masses is strongly dependent on the ultrasound operator’s experience (9). To increase the diagnostic accuracy and repeatability of ultrasonic assessment for adnexal tumors, the International Ovarian Tumor Analysis (IOTA) group created a new ultrasound-based ADNEX (Assessment of Different NEoplasias in the adneXa) model that offers better performance for identifying malignant tumors among adnexal tumors (10). This model can predict the probability of malignancy based on three clinical and six ultrasonic characteristics. Multiple studies have confirmed that the ADNEX model offers better diagnostic performance than previous IOTA models (11–13), with a higher sensitivity (0.98, 95% confidence interval [CI]: 0.93–1.00). However, its specificity was lowest among all models (0.62, 95% CI: 0.55–0.68) (14).

MRI is a helpful tool for distinguishing benign and malignant adnexal tumors. However, the cost and operative time of MRI limit its routine use in the screening of adnexal tumors. According to the European Society of Urogenital Radiology (ESUR) guidelines, MRI is recommended only for masses that cannot be discriminated by ultrasound (15). Previous studies have indicated that the IOTA LR2 model and MRI give comparable results (16–18). However, a multi-center research comparing the abilities of the ADNEX model and MRI for characterizing adnexal tumors has not been reported to our knowledge. The aim of this multi-center study was to compare the diagnostic performances of the ultrasound-based ADNEX model and subjective MRI evaluation for distinguishing benign and malignant adnexal masses. Furthermore, we aimed to assess the diagnostic performance of the combination of the ADNEX model and subjective MRI assessment.

## Material and methods

### Study design and patients

**Figure 1:**
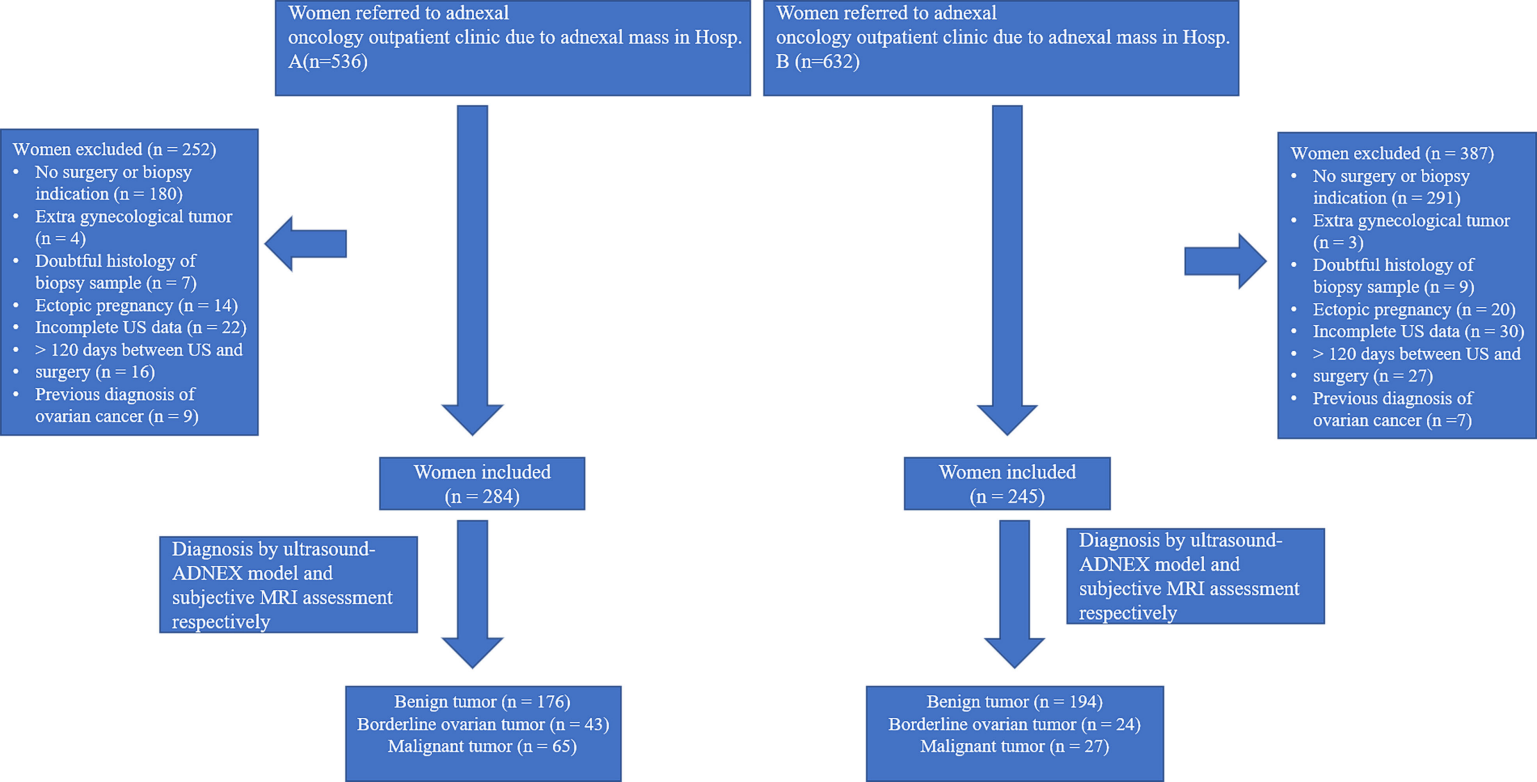
Flowchart of enrollment in study cohort of women diagnosed with adnexal mass in two hospitals.

This multi-center, prospective cohort study was carried out at gynecological oncology center of the First Affiliated Hospital of Chongqing Medical University and the Women and Children’s Hospital of Chongqing Medical University (**Figure 1**). A total of 529 women treated at these two hospitals were enrolled consecutively between October 2019 and April 2022, and their adnexal masses were assessed using both ultrasound and MRI. This study was approved by the institutional ethics committees of the two hospitals, and all patients voluntarily provided informed consent.

The inclusion criteria were as follows: (a) at least one adnexal mass that had been evaluated by ultrasound and MRI examination at either of the two hospitals. The most complicated or largest mass was chosen for the final analysis if bilateral adnexal masses were detected; and (b) planned surgical excision of the mass, as recommended by a gynecological oncologist. The exclusion criteria were as follows: (a) history of ovarian tumor; (b) pregnancy; (c) refusal to undergo ultrasound or MRI examination; and (d) lack of surgical excision of the mass within 120 days after the imaging examinations (16).

All the patients underwent ultrasound and MRI examinations, and the results of the evaluations were recorded simultaneously. The results for serum CA125 were unknown at the time of the ultrasound and MRI examinations.

### Ultrasound acquisition and analysis

All the adnexal masses were assessed by an ultrasound doctor using the IOTA ADNEX model before MRI examination. For all masses, transvaginal ultrasound was performed with a Voluson S6^®^ or Voluson E8^®^ ultrasound system with probe frequencies ranging between 5 and 9 MHz (GE Healthcare Ultrasound, Milwaukee, WI, USA). Transabdominal ultrasound was performed if the masses were so large so that their complete shape could not be seen using transvaginal probes.

The eight variables factored into the calculation were as follows: (a) patient’s age (year); (b) maximum diameter of the lesion (mm); (c) maximum diameter of the largest solid part (mm); (d) > 10 locules in the tumor (yes = 1, no = 0); (e) presence of acoustic shadows (yes = 1, no = 0); (f) number of papillary projections; and (g) presence of ascites (yes = 1, no = 0); (h) Gynecological oncology center (yes=1, no=0). In the IOTA ADNEX assessment for patients, we set 0.15 as the cut-off value of probability of malignancy (POM), and masses were considered malignant if POM >0.15 (19).

### MRI acquisition and analysis

MRI data were preoperatively analyzed subjectively by a radiologist who was blinded to the results of the ADNEX model. MRI examinations were conducted using a 1.5-T MR scanner (Ingenia Ambition; Philips Healthcare, Erlangen, Germany or Signa HD Excite, GE Healthcare, Milwaukee, WI, USA) with a phase-array body coil. The MRI protocol was as follows: axial and sagittal T2-weighted fast spin-echo sequences followed by axial T1-weighted gradient recall echo and diffusion weighted image (DWI: b = 0, 1,000 mm^2^/s) sequences. Then dynamic contrast-enhanced MR images were acquired *via* axial fat-saturated T1-weighted imaging after intravenous injection of a bolus of 0.2 ml/kg gadodiamide as the contrast agent (GE Healthcare).

According to the ESUR guideline (20), the radiologist judged whether the mass was possibly malignant, borderline or benign *via* subjective assessments. MRI data were analyzed by two experienced radiologists. The final MRI results were decided through discussion if the two radiologists originally had conflicting findings for a case.

### Reference standard

After surgery, all excised specimens were examined histologically at one of the two hospitals in the study, and the masses were classified according to the guidelines of the World Health Organization for the classification of tumors (21). For each case, the histopathological diagnosis was considered as the reference standard.

### Statistical analysis

All statistical analyses were using SPSS 25.0 software (IBM, Armonk, NY, USA). The descriptive statistics included mean ± standard deviation for continuous variables and number (percentage) for categorical variables (ultrasound-ADNEX results). The sensitivity, specificity, negative predictive value (NPV), positive predictive value (PPV), and 95% Wilson score confidence intervals were calculated for evaluation of the diagnostic performance of the ultrasound-based ADNEX model and MRI evaluation. Analyses of agreement (percent total agreement) was used to compare the ability of the two methods to detect malignancy. McNemar’s exact χ^2^ test was applied to analyze the differences in discriminatory ability between the two strategies or the two hospitals. We also analyzed the diagnostic efficacy of combining the ADNEX model and subjective MRI assessment. If the two methods produced different results for a case, the mass was then considered a malignant tumor. For analysis of the six variables of the ultrasound-based ADNEX model, one-way analysis of variance (ANOVA) or Mann–Whitney U test (if appropriate) was used to compare variables among benign, borderline, and malignant tumors. Statistical significance was assumed at a level of *P*<0.05 for all comparisons.

## Results

### Diagnoses of patients with adnexal masses

The histologically confirmed diagnoses of 529 patients with at least one adnexal mass are shown in **Table 1**. Overall, 370 (69.9%) masses were benign tumors, and 159 (30.1%) masses were malignant tumors (including 67 borderline and 92 malignant tumors). Malignant tumors were seen in 23.4% (65/278) of patients treated at the First Affiliated Hospital of Chongqing Medical University and in 11% (27/245) treated at the Women and Children’s Hospital of Chongqing Medical University. No statistically significant difference in the diagnostic accuracy rate was detected between the two centers (*P*=0.563, **Table 2**). Although the characteristics of patients treated at the two hospitals were acquired by different researchers, the ultrasound-based ADNEX model and MRI assessments conducted at the two hospitals showed similar diagnostic performance, suggesting the results of this study are generalizable (**Tables 2**, **3**).

**Table 1:** Histological subtypes of adnexal tumors in patients treated in two institutions.

Histology	All(n=529)	By center	
		Hosp. A (n=284)	Hosp. B(n=245)
Benign	370(69.9%)	176 (62.0%)	194 (79.2%)
Teratoma	110(29.7%)	46 (26.1%)	64(33.0%)
Serous cystadenoma	84(22.7%)	50 (28.4 %)	34(17.5%)
Mucinous cystadenoma	49(13.2%)	18(10.2%)	31(16…)
Cystadenofibroma	2(0.5%)	2(1.1%)	0(0)
Fibroma	6(1.6%)	2(1.1%)	4(2.1%)
Brenner tumor	4(1.1%)	3(1.7%)	1(0.5%)
Ovarian torsion	31(8.4%)	20(11.4%)	11(5.7%)
Functional cyst	76(20.5%)	29(16.5%)	47(24.2%)
Other benign lesion	8(2.2%)	6(3.4%)	2(1.0%)
Borderline	67(12.7%)	43(15.1%)	24(9.8%)
Serous borderline tumor	41(61.2%)	29(67.4%)	12(50%)
Mucinous borderline tumor	22(32.8%)	13(30.2%)	9(37.5%)
Other borderline tumor	4(6.0%)	1(2.3%)	3(12.5%)
Malignancy	92(17.4%)	65(22.9%)	27(11.0%)
Serous adenocarcinoma	48(52.2%)	32(49.2%)	16(59.3%)
Clear cell carcinoma	20(21.7%)	14(21.5%)	6(22.2%)
Granulosa cell tumor	7(7.6%)	6(9.2%)	1(3.7%)
Mucinous adenocarcinoma	6(6.5%)	3(4.6%)	3(11.1%)
Sertoli leydig	2(2.2%)	2(3.1%)	0(0.0%)
Ovarian metastasis	9(9.8%%)	8(12.3%)	1(3.7%)

Hosp A: The First Affiliated Hospital of Chongqing Medical University; Hosp B: Women and Children's Hospital of Chongqing Medical University.

**Table 2:** Comparison of the diagnostic performances of the methods at the two institutions.

Imaging method	US-based IOTA ADNEX model		*P*	MRI subjective assessment		*P*	Combination of ADNEX and MRI^a^		*P*
	Hosp. A	Hosp. B		Hosp. A	Hosp. B		Hosp. A	Hosp. B	
Correctly classified	253	222	0.563	237	205	0.945	270	232	0.844
Incorrectly classified	31	23		47	40		14	13	

a Cases of disagreement were classified as malignant. Hosp A: The First Affiliated Hospital of Chongqing Medical University; Hosp B: Women and Children's Hospital of Chongqing Medical University.

### Validation of the IOTA ultrasound-based ADNEX model

**Figure 2:**
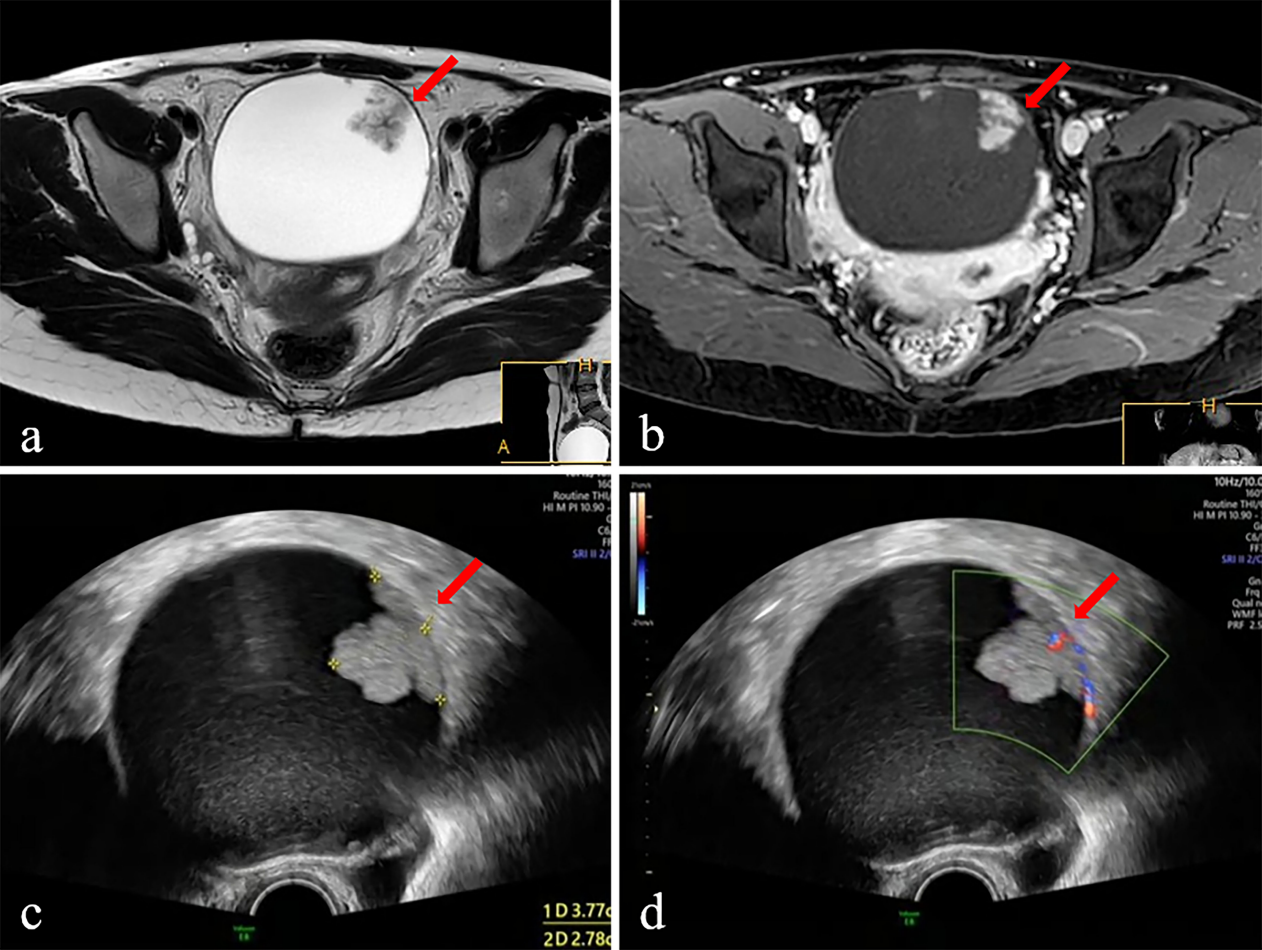
The ultrasound and MR images of a 28-year-old female patient with adnexal mass. The mass was diagnosed borderline tumor preoperatively by ADNEX-US and subjective MRI assessment. **(A)** Axial T 2 WI displays intermediate SI of the solid component (arrow) and high SI of cystic component. **(B)** Postcontrast axial T1-fat-suppressed image shows obvious persistent enhancement of the solid component (arrow) and walls of the cystic component. **(C)** Ultrasound images (Gray scale) displayed an anechoic mass with equal echo of the solid component (arrow). **(D)** Ultrasound images (color Doppler) displayed the solid component of mass has dotted blood flow signal. Surgery was performed, and the diagnosis was confirmed on histopathology as the borderline tumor.

The clinical and sonographic features considered in the ADNEX model are presented in **Table 4**. The patients with malignant tumors were older than those with benign and borderline tumors (both *P*<0.001), and the patients with borderline tumors were older than those with benign tumors (*P*<0.001). Several variables were closely related to the properties of the adnexal masses. For example, the maximum diameter of the lesion and largest solid part of the lesion were greater in cases with malignant tumors than in cases with benign and borderline tumors (all *P*<0.001). However, the maximum diameter of the lesion and largest solid part of the lesion did not differ significantly between benign tumors and borderline tumors (*P*=0.786 and *P*=0.187, respectively). The risk of malignancy was closely related to the presence of ascites (odds ratio [OR]=12.88, 95% confidential interval [CI): 6.45–25.74, *P*<0.001]. However, acoustic shadows were significantly related with benign tumors (OR=7.576, 95% CI: 2.32–24.69, *P*<0.001). The feature of >10 locules was statistically different only between benign and malignant tumors (*P*=0.001). The ADNEX model had a sensitivity of 0.91 (95% CI: 0.85-0.95), specificity of 0.90 (95% CI: 0.87–0.93), PPV of 0.79 (95% CI: 0.73–0.85), and NPV of 0.95 (95% CI: 0.93–0.98; **Table 3**). Among the 529 women, the ADNEX model classified only 54 (10.2%) adnexal tumors incorrectly, including 16 benign tumors as malignant and 38 malignant tumors (including borderline tumors) as benign. **Figures 2** showed representative case.

**Table 3:** Comparison of the diagnostic performances of the ADNEX model and subjective MRI assessment.

Imaging method	Correctly classified		Incorrectly classified		Sensitivity (95% CI)	Specificity (95% CI)	PPV (95% CI)	NPV (95% CI)	Accuracy (95% CI)
	Malignant	benign	Malignant as benign	Benign as malignant					
US-based IOTA ADNEX model									
All	143	332	16	38	0.91 (0.85-0.95)	0.90 (0.87-0.93)	0.79 (0.73-0.85)	0.95 (0.93-0.98)	0.90 (0.87-0.92)
Hosp A	98	155	10	21	0.92 (0.85-0.96)	0.88 (0.83-0.93)	0.88 (0.75-0.89)	0.97 (0.90-0.98)	0.89 (0.85-0.93)
Hosp B	45	177	6	17	0.92 (0.79-0.97)	0.91 (0.87-0.95)	0.80 (0.61-0.84)	0.97 (0.94-0.99)	0.91 (0.87-0.94)
MRI subjective assessment									
All	141	301	18	69	0.89 (0.84-0.94)	0.81 (0.77-0.85)	0.67 (0.61-0.74)	0.94 (0.92-0.97)	0.84 (0.80-0.87)
Hosp A	96	141	12	35	0.89 (0.83-0.95)	0.80 (0.74-0.86)	0.73 (0.66-0.81)	0.92 (0.88-0.97)	0.83 (0.79-0.88)
Hosp B	45	160	6	34	0.88 (0.79-0.97)	0.82 (0.77-0.88)	0.57 (0.46-0.68)	0.96 (0.94-0.99)	0.84 (0.79-0.88)
Combination of ADNEX and MRI^a^									
All	154	346	5	24	0.97 (0.94-1.00)	0.94 (0.91-0.96)	0.87 (0.82-0.92)	0.99 (0.97-1.00)	0.95 (0.93-0.97)
Hosp A	105	165	3	11	0.97 (0.94-1.00)	0.93 (0.90-0.97)	0.91 (0.85-0.96)	0.98 (0.96-1.00)	0.95 (0.93-0.98)
Hosp B	49	183	2	11	0.96 (0.91-1.00)	0.94 (0.91-0.98)	0.82 (0.71-0.92)	0.99 (0.97-1.00)	0.95 (0.92-0.97)

a Cases of disagreement were classified as malignancy. Hosp A: The First Affiliated Hospital of Chongqing Medical University; Hosp B: Women and Children's Hospital of Chongqing Medical University.

Malignant tumor included the borderline tumor. Malignant include borderline tumor.

**Table 4:** Sonographic features of adnexal masses in 529 women treated in two institutions.

Variables included in IOTA ADNEX model	Benign	borderline	Malignant	*P*
Age (years)	47.4±13.4	51.0±13.6	56.5±11.3	<0.001
Ascites	11(3.0)	2(3.0)	43(46.7)	<0.001
Maximal diameter of the lesion (mm)	118.1±58.8	122.2±46.6	157.4±73.4	<0.001
Maximal diameter of the largest solid part (mm)	34.3±29.2	55.6±28.0	93.0±48.9	<0.001
>10 locules	52(14.1)	11(16.4)	28(30.4)	0.001
Number of papillary projections				NA
0	346(93.5)	45(67.2)	75(81.5)	
1	10(2.7)	12(17.9)	7(7.6)	
2	4(1.1)	3(4.5)	0(0)	
3	6(1.6)	0(0)	3(3.3)	
>3	4(1.1)	7(10.4)	7(7.6)	
Acoustic shadows	89(24.1)	2(3.0)	1(1.1)	<0.001

Data are given as mean ± SD or n (%). Groups compared using McNemar’s exact χ2, one-way analysis of variance or Kruskal–Wallis test, if appropriate. Max, maximum; NA, not applicable.

### Subjective MRI assessment results

For distinguishing malignant tumors, including borderline tumors, from benign adnexal tumors, MRI had a sensitivity of 0.89 (95% CI: 0.84–0.94), specificity of 0.81 (95% CI: 0.77–0.85), PPV of 0.67 (95% CI: 0.61–0.74), and NPV of 0.94 (95% CI: 0.92–0.97; **Table 3**). Among the 529 cases, MRI classified 87 (16.4%) adnexal tumors incorrectly, including 18 malignant tumors (including borderline tumors) as benign and 69 benign tumors as malignant. **Figures 2** showed representative case.

### Comparison of the diagnostic performances of the ADNEX model and subjective MRI assessment

The results for the preoperative diagnostic accuracy and agreement of the two methods are shown in **Table 5**. Good total agreement (84.9%) between the ADNEX model and subjective MRI assessment was observed, but poor agreement between the ADNEX model and MRI was observed for borderline tumors (67.2%). From the comparison of diagnostic performance, the sensitivity of the ADNEX model (0.91; 95% CI: 0.85-0.95) for detecting malignant tumors, including borderline tumors, was similar to that of MRI (0.89; 95% CI: 0.84–0.94; *P*=0.717; **Table 4**). However, the specificity of the ADNEX model (0.90; 95% CI: 0.87–0.93) was higher than of MRI (0.81; 95% CI: 0.77–0.85; *P*=0.001; **Table 4**). The accuracy of the ADNEX model (0.90; 95% CI: 0.87–0.92) did not differ significantly from that of MRI assessment (0.84; 95% CI: 0.80-0.87, *P*=0.059, **Table 5**). However, when we compared the agreement rate between the ADNEX model and MRI for borderline tumors, the ADNEX model showed superior accuracy compared with MRI (*P*<0.001, **Table 5**). No statistically significant differences were detected between the two methods for benign and malignant tumors (*P*=0.721 and *P*=0.246 respectively, **Table 5**). When we combined the ADNEX model with subjective MRI assessment, the sensitivity increased to 0.97 (95% CI: 0.94–1.00) and the specificity increased to 0.94 (95% CI: 0.91–0.96), and these values were significantly higher than those for either the ADNEX model (*P*=0.013 and *P*=0.001, respectively) or MRI (*P*=0.005 and *P*<0.001) alone.

**Table 5:** Diagnostic agreement and comparison of agreement rates between the ADNEX model and MRI with adnexal tumor histology as reference standard.

Histology	n	Discriminating between different types of tumors				*p*	Agreement analysis				Agreement (%)
		US-ADNEX model		MRI subjective assessment			US-ADNEX correct		US-ADNEX incorrect		
		Correctly classified	Incorrectly classified	Correctly classified	Incorrectly classified		MRI correct	MRI incorrect	MRI correct	MRI incorrect	
All	529	475	54	455	74	0.059	425	50	30	24	84.9%
Benign	370	332	38	329	41	0.721	308	24	21	17	87.8%
Borderline	67	64	3	42	25	<0.001	42	22	0	3	67.2%
Malignant	92	79	13	84	8	0.246	75	4	9	4	85.9%

## Discussion

The IOTA ultrasound-based ADNEX model performed well in distinguishing malignant and benign adnexal masses using data obtained in two hospitals in China, especially for borderline tumors, even though CA125 level data were not included in this study. Although CA125 is one of the clinical variables (www.iotagroup.org/adnexmodel/), the applications allow risk calculation even without information on serum CA-125 level despite the decrease in performance and it is important for good discrimination between stage II-IV cancer and stage I and secondary metastatic cancer in the ultrasound-based ADNEX model (10). Besides, previous studies also demonstrated that the CA125 level had no significant impact on the diagnostic accuracy of the ADNEX model (22–24). This is because CA125 is not a specific marker for ovarian cancer, and it can be increased in cases with benign lesions, such as endometriosis and uterine fibroids (25, 26). Human epididymal protein-4 (HE-4) has been identified as a new tumor marker for ovarian cancer (27), and research has verified that HE-4 is more valuable than CA125 for ovarian cancer (28). As a result, the ADNEX model may be further optimized for the diagnosis of adnexal masses in the future.

In the present study, the diagnostic performance of the two methods showed no statistically significant difference between the two hospitals, suggesting good repeatability of these methods in two institutions. In addition, the diagnostic performance for the ADNEX model was similar to that of the expert US examiners’ subjective assessment in the analysis of 3511 adnexal masses (29). These observations indicate that the IOTA ultrasound-based ADNEX model is a widely applicable tool in different populations and institutions to assist sonographers, gynecologists, and even non-professional doctors with various training backgrounds and levels of experience in the diagnosis of adnexal tumors. However, the sensitivity and specificity of the ADNEX model in our study was lower than that calculated in by Valentin et al. (29), whereas the specificity in our study was higher than that in other studies (11, 14). This may be related to differences in the study samples, but another reason could be use of the cut-off value of 0.15 for the ADNEX model results. Because Huang X et al. have found that the cut-off value of 0.15 for the ADNEX model had high diagnostic accuracy in identifying ovarian malignant tumor (19).

We noted obvious differences in the maximum diameter of lesions and the largest solid component of tumors in the present study, but these findings differed from those in a previous study (11). Moreover, in our study, the ultrasound feature of acoustic shadowing was applied as a predictive criterion for benign adnexal tumors and the risk of malignancy was closed related with the presence of ascites, findings which were similar to those of the previous study (11). These results indicate the importance of the features of acoustic shadowing and ascites.

The present study showed good agreement between the ADNEX model and MRI assessment. Additionally, the sensitivity of the ADNEX model was similar to that of MRI. However, the ADNEX model had a higher specificity, suggesting that the ADNEX model provided fewer false-positive cases compared with MRI. Among the benign, borderline and malignant tumors, the agreement rate between the ADNEX model and MRI was lowest for borderline tumors (only 67.2%), suggesting that the diagnostic accuracy of the ADNEX model for borderline tumors was superior to that of MRI. Although previous studies have reported characteristics of borderline tumors, few parameters can reliably differentiate borderline tumors from benign tumors on MRI (30, 31). Perhaps this was a reason that the specificity of the ADNEX model was higher than that of MRI. As a result, the ADNEX model may play an additional important role in determining the appropriate surgical management before operation and can be helpful to promote optimal patient management in the future due to its good diagnostic accuracy rate.

In the current study, the ADNEX model classified 54 (10%) adnexal tumors incorrectly. A collaborative analysis of IOTA studies reported that only a small portion (approximately 7%) of adnexal masses cannot be accurately classified preoperatively, even when subjective ultrasound assessment is performed by an experienced sonographer (29). However, this collaborative analysis aimed to discriminate between benign and malignant tumors using a logistic regression (LR) model only for masses that were deemed unclassifiable by the sonographer. This is likely the reason that the rate of inaccurate classification was higher in our study than in the previous analysis.

The combination of the ADNEX model and MRI provided improved accuracy for the preoperative diagnosis of adnexal tumors than either method alone, likely because subjective MRI assessment underestimated the risk of malignancy. In the present study, the greatest sensitivity and specificity also were obtained by combining the ADNEX model and subjective MRI assessment. Therefore, to decrease the risk of misclassification, combination of both imaging strategies should be recommended for preoperative assessment of adnexal masses.

This study has some limitations to consider. First, the numbers of enrolled patients and institutions were small for a multi-center study. Although the ADNEX model demonstrated greater specificity than MRI in the present study, these limitations likely affected the diagnostic performance of both methods. Second, all MRI examinations were performed on a 1.5T MR system, and we did not compare differences between results obtained with a 3.0T MRI system and the ADNEX model. Third, the ADNEX model was not used for clinical management, and therefore, the influence of this model on patient management is unknown. Fourth, because the ADNEX model is still not commonly used, it remains unfamiliar to many clinicians. Moreover, it is still under modification in China, especially for use in primary hospitals.

In conclusion, the IOTA ultrasound-based ADNEX model is as sensitive as subjective MRI assessment for distinguishing adnexal tumors, but has a higher specificity compared with MRI and a higher accuracy rate for borderline tumors compared with benign and malignant tumors. These findings reveal that the ADNEX model is a reliable points-scoring system for the preoperative diagnosis of adnexal mass. We recommend the addition of the ADNEX model, either alone or in combination with MRI, for preoperative assessment of adnexal masses.

## Data availability statement

The original contributions presented in the study are included in the article/supplementary material. Further inquiries can be directed to the corresponding author.

## Ethics statement

The studies involving human participants were reviewed and approved by Ethics Committee of the First Affiliated Hospital of Chongqing Medical University and Women and Children’s Hospital of Chongqing Medical University. Written informed consent to participate in this study was provided by the participants’ legal guardian/next of kin. Written informed consent was obtained from the individual(s), and minor(s)’ legal guardian/next of kin, for the publication of any potentially identifiable images or data included in this article.

## Author contributions

Conception and design: YH, BC, HD, BS, ZX, JL, WT, FL. Analysis and interpretation: YH, BC. Data collection: HD, BS, ZX, JL, WT. Writing the article: YH, FL. Critical revision of the article: YH, BC, HD, BS, ZX, JL, WT, FL. Final approval of the article: YH, BC, HD, BS, ZX, JL, WT, FL. Statistical analysis: YH, BC Obtained funding: FL. Overall responsibility: FL. All authors contributed to the article and approved the submitted version.
